# Serum α-Klotho and fibroblast growth factor 23 levels are not associated with non-proliferative diabetic retinopathy in type 1 diabetes mellitus

**DOI:** 10.1038/s41598-024-54788-1

**Published:** 2024-02-19

**Authors:** Can Oner, Burcu Dogan, Sabah Tuzun, Asiye Ekinci, Gunes Feyizoglu, Banu Isbilen Basok

**Affiliations:** 1 Department of Family Medicine, Health Sciences University Kartal Dr Lutfi Kirdar City Hospital, Istanbul, Turkey; 2 Department of Family Medicine, Health Sciences University Gulhane Training and Research Hospital, Ankara, Turkey; 3 Department of Family Medicine, Health Sciences University Haseki Sultangazi Training and Research Hospital, Istanbul, Turkey; 4 Department of Ophtalmology, Health Sciences University Bursa Yuksek Ihtisas Training and Research Hospital, Bursa, Turkey; 5 Department of Internal Medicine, Goztepe Prof Dr Suleyman Yalcın City Hospital, Istanbul, Turkey; 6 Department of Medical Biochemistry, Health Sciences University Tepecik Training and Research Hospital, Izmir, Turkey

**Keywords:** α-Klotho, FGF 23, Diabetic retinopathy, Diabetes mellitus, Biochemistry, Endocrinology, Molecular medicine

## Abstract

Diabetic retinopathy is a commonly observed cause of blindness and is a common problem in individuals with diabetes. Recent investigations have showed the capability of serum α-Klotho and FGF 23 in mitigating the effects of diabetic retinopathy. This study aimed to discover the correlation between FGF 23, α-Klotho, and diabetic retinopathy in type 1 diabetics. This case–control study included 63 diabetic patients and 66 healthy controls. Following an overnight duration of fasting, morning blood samples were taken from both the patient and the control groups. The serum concentrations of α-Klotho and FGF 23 were quantified. An experienced ophthalmologist inspected the retinopathy. All participants in this study have moderate non-proliferative retinopathy. A p value under 0.05 was considered statistically significant. The mean α-Klotho level for retinopathic diabetic patients was 501.7 ± 172.2 pg/mL and 579.6 ± 312.1 pg/mL for non-retinopathic diabetic patients. In comparison, α-Klotho level of the control group was 523.2 ± 265.4 pg/mL (p = 0.531). The mean of FGF 23 level did not demonstrate a significant difference (p = 0.259). The mean FGF 23 level were 75.7 ± 14.0 pg/mL, 74.0 ± 14.8 pg/mL and 79.3 ± 14.4 pg/mL in groups, respectively. In conclusion, there was no significant difference in FGF 23 and α-Klotho levels between type 1 diabetics with and without retinopathy when compared to the control group.

## Introduction

Diabetes mellitus, a chronic, multisystem disease, can lead to various complications and comorbidities, one of which is diabetic retinopathy. Diabetic retinopathy is one of the leading causes of blindness and visual impairment in adults and affects approximately 30–40% of all diabetic patients^1^. The occurrence of diabetic retinopathy is the result of a complex mechanism. Hyperglycemia plays a key role in this process. It disrupts metabolic pathways and increases oxidative stress, leading to vascular endothelial dysfunction^2^. This dysfunction leads to increased vascular permeability and retinal ischemia. Increased permeability leads to macular oedema, while retinal ischemia triggers angiogenesis and retinal neovascularization^3^. Recent studies suggest novel molecular processes that may influence the development and severity of diabetic retinopathy. Two molecules of interest on which these studies have focused are α-Klotho and fibroblast growth factor-23 (FGF 23)^4–6^.

Klotho protein was first discovered in 1997 as an anti-aging factor. It comprises three isoforms: α-Klotho, β-Klotho and γ-Klotho. These isoforms have different tissue expression patterns and biological functions^7^. α-Klotho is mainly found in the renal tubular epithelium, choroid plexus in the brain, and secreted in the blood^8^. It has been shown to act as a co-receptor for FGF 23, which helps to regulate calcium and phosphorus metabolism^7^. Studies have shown that α-Klotho inhibits insulin signaling^9^, regulates glucose and fat metabolism^10^ and plays a role in the central regulation of energy balance^11^. Recent studies have shown that diabetic patients have reduced serum Klotho levels^12–14^.

Novel studies suggests that α-Klotho has a positive impact on maintaining retinal health in diabetic retinopathy^15^. In vivo studies, it was found that Klotho is present in all layers of the retina and that its deficiency leads to weakened retinal signaling and altered synaptic function in mice^16^. Additionally, decreased levels of α-Klotho in the lens of diabetic rats results in a decline in antioxidant defence and contribute to increased inflammation^17^. Studies have also demonstrated that the Klotho protein plays a crucial role in preserving the viability of the retinal pigment epithelium by promoting the growth of mitochondria and reducing oxidative stress, inhibiting endothelial cell apoptosis, enhancing autophagy, and counteracting epithelial mesenchymal transition^4,18,19^. Furthermore, it indirectly suppresses the secretion of vascular endothelial growth factor (VEGF) from the retinal pigment epithelium, preventing neovascularization, a critical step in the development of diabetic retinopathy, and helps to maintain the normal structure of blood vessels in the choroidal layer^20^.

Many laboratories and clinical studies have suggested that Klotho could be a potential solution for diabetic retinopathy^4,15^. However, there have been very few studies that have explored the connection between serum α-Klotho, FGF 23 levels and diabetic retinopathy, particularly in type 1 diabetes. This study aimed to investigate the correlation between serum α-Klotho, FGF 23 levels and non-proliferative diabetic retinopathy in individuals with type 1 diabetes.

## Methods

This case–control study was conducted at the Istanbul Medeniyet University Goztepe Training and Research Hospital. It involved 63 type 1 diabetic patients and 66 healthy controls. Type 1 diabetic patients, who were pregnant, had chronic inflammation or infection, hypertension and eGFR ≤ 60 mL/min, and non-diabetic with renal disease were excluded from the study. As a control group, individuals without diabetes who had fasting plasma glucose levels of < 100 mg/dL (following overnight fasting), eGFR of ≥ 60 mL/min, and had no prior history of diabetes, renal disease, cardiovascular diseases including, hypertension and dyslipidemia were randomly enrolled in the study. Written informed consent was obtained from all participants before enrolling in the study. The study was approved by the institutional ethics committee (14.02.2012/19/J-29/e). The procedures followed in this study were conducted in strict accordance with the relevant guidelines and regulations.

A professional ophthalmologist assessed retinopathy using direct fundoscopy while the patients were in a state of mydriasis. Fundus examinations under mydriasis by trained ophthalmologists are considered the standard in most studies. Diabetic retinopathy was classified according to The International Clinical Diabetic Retinopathy (ICDR) Severity Scale which is convenient and easy to use in everyday clinical practice. According to ICDR diabetic retinopathy is classified into five groups: no apparent retinopathy, mild non-proliferative diabetic retinopathy, moderate non-proliferative diabetic retinopathy, severe non-proliferative diabetic retinopathy and proliferative diabetic retinopathy^22^. In this study, patients with proliferative diabetic retinopathy and mild/severe non-proliferative diabetic retinopathy were excluded. All the patients included have moderate-level non-proliferative retinopathy. Morning blood samples were collected from both the patient and control groups after an overnight fasting period. After allowing the blood samples to clot at most 2 h at room temperature, they were centrifuged at 1500 g for 10 min to obtain serum matrices. The serum samples were aliquoted and stored at -80 °C until the analysis. Serum Klotho levels were determined by the ELISA method via the Klotho ELISA kit (Cusabio Biotech Co., Ltd., P.R. China). The minimum detectable level of the Klotho test was 0.156 ng/mL and the sensitivity was 0.039 ng/mL. The values are converted from ng/mL to pg/mL before analysis. The intra- and inter-assay coefficients of variability of the test were < 8 and < 10%, respectively. Serum FGF 23 levels were measured using a commercial ELISA kit (EMD Millipore Corp., USA) by following the manufacturer’s instructions. The limit of sensitivity of the assay was 3.5 pg/mL. The intra- and inter-assay coefficients of variability of the FGF 23 test at the concentrations of 75.7 and 78.5 pg/mL were 11.2 and 2.45%, respectively. The statistical analysis was conducted using SPSS 21.0 and G Power Statistical Software for power analysis and sample size. Data were expressed as mean ± standard deviation, median (minimum–maximum), and percent (%) where appropriate. Student's t-test and one-way ANOVA were used for numerical data with a normal distribution, while Mann–Whitney U and Kruskal–Wallis tests were used for non-normally distributed variables. The chi-square test was used for the comparison of categorical data. Pearson's correlation was used for variables with a normal distribution, while Spearman's rho correlation was used for non-normally distributed variables. A p value under 0.05 was considered statistically significant.

## Results

The study involved 129 participants; 63 (48.8%) were with type 1 diabetics and 66 (51.2%) were controls. The mean age of the whole group was 34.5 ± 9.3 years and half of the sample was male at 50.4% (n = 65) while 49.6% (n = 64) were female. Both the case and control groups possessed similar general features, except for the HbA1c and fasting glucose levels as shown in Table 1. The diabetic group was further divided into two subgroups. The first subgroup consisted of type 1 diabetes patients with retinopathy (73.0%, n = 46), and the second subgroup included type 1 diabetics without retinopathy (27.0%, n = 17). The general features of the diabetic group were summarized in Table 2.

**Table 1:** General features of the study groups.

	Type 1 diabetics (n = 63)	Controls (n = 66)	p
Age (years)	34.8 ± 9.5	34.2 ± 9.2	0.738*
Gender (%)			
Male	55.6 (35)	45.5 (30)	0.292**
Female	44.4 (28)	54.5 (36)	
BMI (kg/m^2^)	25.7 ± 9.2	26.8 ± 9.0	0.485*
HbA1c (%)	9.0 ± 2.0	5.2 ± 0.9	< 0.001*
Fasting glucose (mg/dL)	205.4 ± 92.5	88.5 ± 14.7	< 0.001*
Creatinine (mg/dL)	0.8 ± 0.2	0.9 ± 0.2	0.250*
HDL (mg/dL)	51.8 ± 14.0	50.4 ± 15.0	0.550*
LDL (mg/dL)	125.5 ± 45.0	112.4 ± 30.8	0.058*
Triglyceride (mg/dL)	134.8 ± 135.8	107.0 ± 64.8	0.140***
TSH (mIU/L)	2.8 ± 4.6	1.8 ± 1.0	0.102***

*BMI* body mass index, *HDL* high density lipoprotein, *LDL* low density lipoprotein, *TSH* thyroid stimulating hormone.

*Student t test **Chi Square test ***Mann Whitney U test.

**Table 2:** General features of type 1 diabetics with and without retinopathy.

	Retinopathic Type 1 diabetics (n = 46)	Non-retinopathic Type 1 diabetics (n = 17)	p
Age (years)	34.4 ± 9.4	35.8 ± 10.1	0.603*
Gender (%)			
Male	63.0 (29)	35.3 (6)	0.085**
Female	37.0 (17)	64.7 (11)	
BMI (kg/m^2^)	26.4 ± 10.5	23.7 ± 4.0	0.321*
HbA1c (%)	9.2 ± 1.9	8.5 ± 2.2	0.217*
Fasting glucose (mg/dL)	211.1 ± 100.0	190.1 ± 65.9	0.430*
Creatinine (mg/dL)	0.9 ± 0.2	0.8 ± 0.1	0.224*
HDL (mg/dL)	52.3 ± 15.6	50.5 ± 8.6	0.659*
LDL (mg/dL)	126.5 ± 46.5	122.8 ± 42.0	0.775*
Triglyceride (mg/dL)	145.6 ± 156.2	105.4 ± 39.8	0.301*
TSH (mIU/L)	3.0 ± 5.3	2.0 ± 1.8	0.437*

*BMI* body mass index, *HDL* high density lipoprotein, *LDL* low density lipoprotein, *TSH* thyroid stimulating hormone.

*Mann Whitney U test ** Chi Square test.

**Figure 1:**
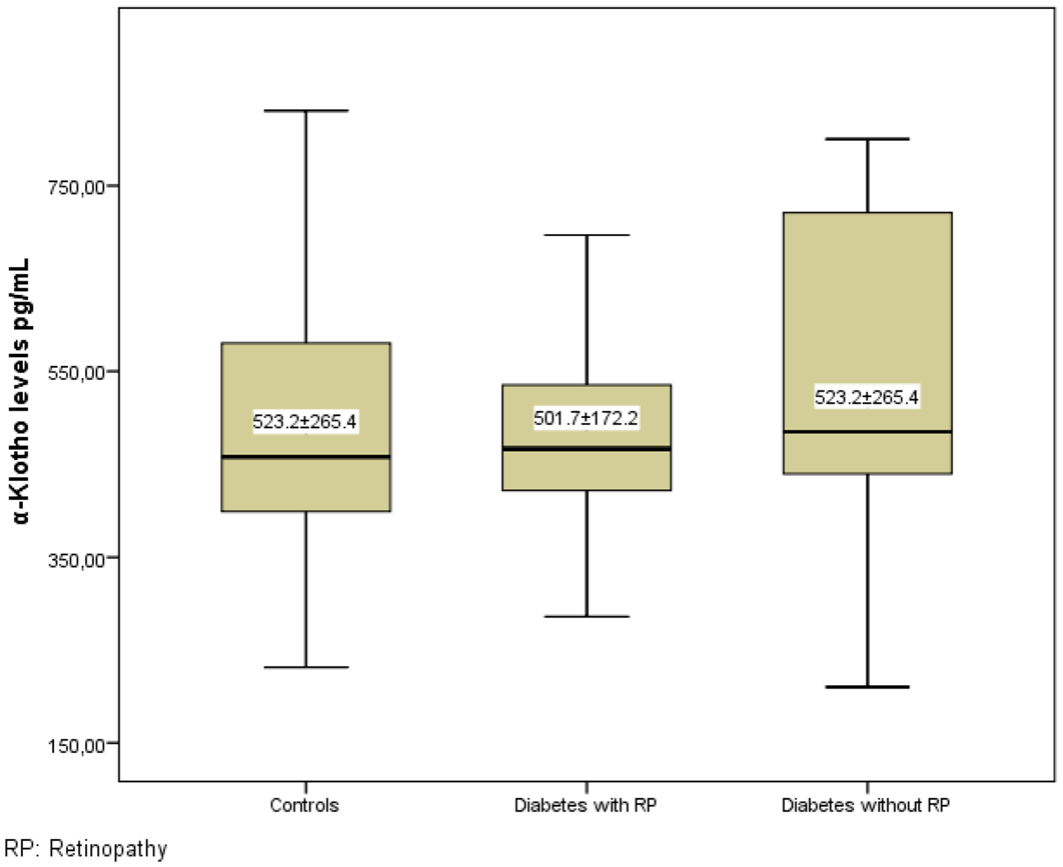
Comparison of serum α-Klotho levels in three groups.

The mean α-Klotho level of retinopathic diabetic patients was 501.7 ± 172.2 pg/mL. Non-retinopathic diabetic patients had a mean α-Klotho level of 579.6 ± 312.1 pg/mL, while the control group had a mean α-Klotho level of 523.2 ± 265.4 pg/mL. The results of the comparison among the groups showed non-significant differences (p = 0.531) (Fig. 1).

**Figure 2:**
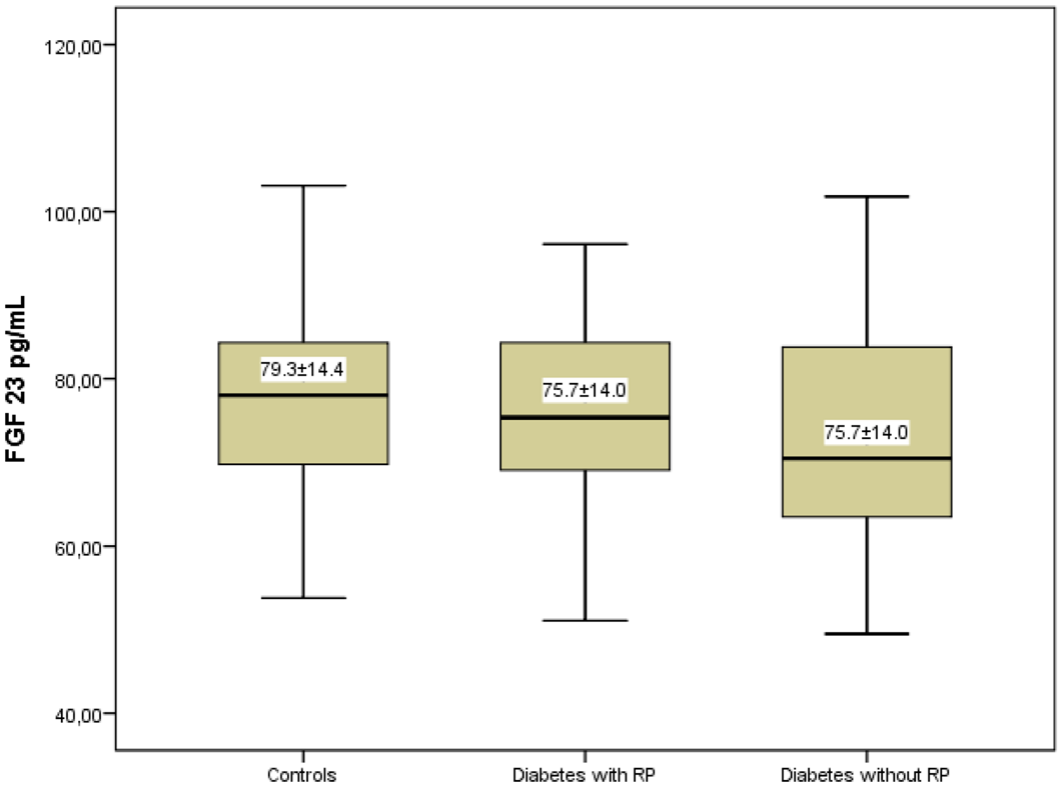
Comparison of serum FGF 23 levels in three groups.

The mean FGF 23 level of retinopathic diabetic patients was 75.7 ± 14.0 pg/mL. Non-retinopathic diabetic patients had a mean FGF 23 level of 74.0 ± 14.8 pg/mL, while the control group had a mean FGF 23 level of 79.3 ± 14.4 pg/mL. When comparing the groups, there were no significant differences (p = 0.259) (Fig. 2).

Table 3 shows the correlation between α-Klotho and FGF 23 levels with age, body mass index (BMI), and metabolic parameters in both groups. A weak positive correlation was found between triglyceride and α-Klotho levels (R = 0.092; r = 0.304; p = 0.040) only in diabetic patients.

**Table 3:** Correlation of α-Klotho and FGF 23 levels with demographic and laboratory data in both groups.

	Diabetics (n = 63)		Controls (n = 66)	
	α-Klotho (pg/mL)*	FGF 23 (pg/mL)*	α-Klotho (pg/mL)*	FGF 23 (pg/mL)*
Age (year)	r = -0.119 p = 0.353	r = 0.025 p = 0.847	r = 0.136 p = 0.653	r = 0.117 p = 0.350
BMI (kg/m^2^)	r = 0.094 p = 0.476	r = 0.100 p = 0.446	r = 0.014 p = 0.912	r = -0.195 p = 0.129
Fasting blood glucose (mg/dL)	r = -0.142 p = 0.347	r = 0.161 p = 0.284	r = -0.048 p = 0.700	r = 0.007 p = 0.955
HbA1c	r = -0.022 p = 0.884	r = 0.053 p = 0.728	r = 0.039 p = 0.756	r = -0.095 p = 0.447
LDL (mg/dL)	r = 0.108 p = 0.475	r = 0.095 p = 0.531	r = 0.085 p = 0.497	r = 0.148 p = 0.236
HDL (mg/dL)	r = -0.007 p = 0.961	r = -0.188 p = 0.212	r = -0.051 p = 0.689	r = 0.075 p = 0.553
Triglyceride (mg/dL)	r = 0.304 p = **0.040**	r = 0.004 p = 0.977	r = 0.088 p = 0.484	r = 0.121 p = 0.339

*BMI* body mass index, *HDL* high density lipoprotein, *LDL* low density lipoprotein.

*Pearson correlation.

## Discussion

We found no significant difference in serum α-Klotho and FGF 23 levels between retinopathic type 1 diabetic patients and the control group. There was a weak positive correlation between triglyceride and serum α-Klotho levels in diabetic participants. The relationship between α-Klotho and diabetic retinopathy has recently been shown only in vivo studies and there is a lack of studies investigating this connection in clinical settings. A few studies focusing on individuals with type 2 diabetes, mainly compared individuals with retinopathy to those without, but the severity of retinopathy was not standardized. To the best of our knowledge, there is no study conducted in type 1 diabetics with non-proliferative retinopathy similar to ours, making it difficult to compare our findings.

We determined no significant difference in α-Klotho levels between individuals with type 1 diabetes and the controls. However, the group of type 1 diabetics without retinopathy had the highest α-Klotho levels, while the group with retinopathy had the lowest. Corcillo et al. followed type 2 diabetic patients for 44 months and they showed that 57% of the patients developed retinopathy. The patients who had lower levels of Klotho were more likely to progress to retinopathy. The authors concluded that reducing circulating Klotho levels by half, increased the risk of retinopathy progression by 44%^22^. Another study which compared type 2 diabetic patients and healthy participants, demonstrated that the diabetic patients had lower serum Klotho levels, which decreased as they progressed from non-retinopathy to non-proliferative and finally to proliferative retinopathy subgroups^19^. In our study, we did not have a proliferative retinopathy group, but we observed lower mean serum α-Klotho levels in the retinopathy group. Słomiński et al. conducted a study in type 1 diabetics and age-matched controls. They revealed that the Klotho gene variant was less common in the retinopathy group compared to uncomplicated diabetic patients. This suggests that high levels of Klotho protein activity may protect against microvascular inflammation and endothelial dysfunction that contribute to retinopathy^23^.

Our study revealed a non-significant difference in serum FGF 23 levels between groups. Despite this, the control group had the highest levels of serum FGF 23. Mehta et al. studied the relationship between FGF 23 levels and retinopathy in patients with chronic renal failure. They came to a similar conclusion that there was no association between serum FGF 23 levels and retinopathy, regardless of the presence of diabetes, hypertension, or other cardiovascular risk factors^24^. However, other studies suggest that FGF 23 and retinopathy may be linked in patients with proliferative diabetic retinopathy^25,26^. The conflicting results may be due to differences between groups and a discrepancy between cellular and clinical studies^24^. Considering the fact that the Klotho protein is a cofactor for FGF 23, it may be logical that levels of both proteins do not differ between the groups^27^.

We found a weak positive correlation between serum α-Klotho and triglyceride levels in type 1 diabetic patients, contrary to previous research. The similarity in lipid parameters between our study group consisting of type 1 diabetics and the controls may have influenced these results. There is no previous research on this relationship in type 1 diabetic patients. Some studies suggest that, especially β-Klotho levels affect lipid hemostasis^28^. Recent studies have found a negative correlation between Klotho concentrations and plasma triglyceride levels^29–31^. In an experimental study, administering α-Klotho to obese mice improved lipid profiles and reduced fatty liver disease^32^. Considering the therapeutic potential of the FGF-Klotho endocrine axis in multiple systems, it is reasonable to believe that Klotho may improve plasma lipid levels^33^.

There are limitations of our study. First, the study was conducted at a single centre and the sample size of the study was small. Second, although we used serum α-Klotho as a biomarker, these levels may not reflect the Klotho protein concentration in tissue. Moreover, Klotho may be affected by circadian rhythm and temporal variation^34,35^. Third, although we accounted for several potential confounding factors, unmeasured and unknown factors may still play a role in the associations because of the complex nature of retinopathy development. Finally, the nephropathic status of the patients was not addressed in the study. There are reports declaring that development of nephropathy and retinopathy in diabetes mellitus follows similar pathways^15^. Therefore, it is not possible to get a complete picture. On the other hand, this study has several strengths. It is conducted as a clinical study, and the results may be valuable and important for clinicians and diagnosticians. Additionally, the retinopathy levels of diabetic patients were standardized, ensuring consistency and reliability in the results.

In conclusion, α-Klotho and FGF 23 levels are not associated with non-proliferative diabetic retinopathy in type 1 diabetes mellitus. This study was clinical in nature and did not investigate the underlying mechanism of the Klotho/FGF23 axis on retinopathy progression. Our results provide a basis for further research in this novel field, but additional clinical research is needed to confirm these assumptions.

## Data Availability

The datasets generated and analyzed during the current study available from the corresponding author on reasonable request.
